# Testing Raman spectroscopy as a diagnostic approach for Lyme disease patients

**DOI:** 10.3389/fcimb.2022.1006134

**Published:** 2022-10-27

**Authors:** Nicolas K. Goff, Tianyi Dou, Samantha Higgins, Elizabeth J. Horn, Rohini Morey, Kyle McClellan, Dmitry Kurouski, Artem S. Rogovskyy

**Affiliations:** ^1^ Department of Biochemistry and Biophysics, Texas A&M University, College Station, TX, United States; ^2^ Lyme Disease Biobank, Portland, OR, United States; ^3^ Department of Veterinary Pathobiology, School of Veterinary Medicine and Biomedical Sciences, Texas A&M University, College Station, TX, United States

**Keywords:** Raman spectroscopy, Lyme borreliosis, *Borrelia*, PLS-DA, diagnostics, test

## Abstract

Lyme disease (LD), the leading tick-borne disease in the Northern hemisphere, is caused by spirochetes of several genospecies of the *Borreliella burgdorferi sensu lato* complex. LD is a multi-systemic and highly debilitating illness that is notoriously challenging to diagnose. The main drawbacks of the two-tiered serology, the only approved diagnostic test in the United States, include poor sensitivity, background seropositivity, and cross-reactivity. Recently, Raman spectroscopy (RS) was examined for its LD diagnostic utility by our earlier proof-of-concept study. The previous investigation analyzed the blood from mice that were infected with 297 and B31 strains of *Borreliella burgdorferi sensu stricto* (*s.s.*). The selected strains represented two out of the three major clades of *B. burgdorferi s.s.* isolates found in the United States. The obtained results were encouraging and prompted us to further investigate the RS diagnostic capacity for LD in this study. The present investigation has analyzed blood of mice infected with European genospecies, *Borreliella afzelii* or *Borreliella garinii*, or *B. burgdorferi* N40, a strain of the third major class of *B. burgdorferi s.s.* in the United States. Moreover, 90 human serum samples that originated from LD-confirmed, LD-negative, and LD-probable human patients were also analyzed by RS. The overall results demonstrated that blood samples from *Borreliella*-infected mice were identified with 96% accuracy, 94% sensitivity, and 100% specificity. Furthermore, human blood samples were analyzed with 88% accuracy, 85% sensitivity, and 90% specificity. Together, the current data indicate that RS should be further explored as a potential diagnostic test for LD patients.

## Introduction

Lyme disease (LD) is an emerging infectious disease, which was first recognized in the 1970s near Lyme, Connecticut (Steere et al., 1977). LD is caused by the spirochetal bacterium of the *Borreliella burgdorferi sensu lato* (*B. burgdorferi s*.*l*.) complex. It should be mentioned that, based on a number of molecular and phenotypic characteristics, the old genus name of the complex genospecies, *Borrelia*, was recently changed (although not without some scientific debate (Adeolu and Gupta, 2014; Barbour et al., 2017; Margos et al., 2017a; Margos et al., 2017b; Margos et al., 2018; Gupta, 2019; Margos et al., 2020; Barbour and Gupta, 2021)) to *Borreliella* in order to distinguish between *B. burgdorferi s*.*l*. and relapsing fever *Borrelia* spirochetes. The enzootic cycle of the LD spirochete is maintained by *Ixodes* ticks and mammalian reservoirs (Steere et al., 2016; Stone et al., 2017). The ixodid ticks, which are responsible for transmission of the LD pathogen in the United States, are *Ixodes scapularis* and *Ixodes pacificus*, whereas in Europe and Asia these are, respectively, *Ixodes ricinus* and *Ixodes persulcatus* (Rand et al., 2003).

The three main genospecies of *B. burgdorferi s.l., Borreliella afzelii* (*B. afzelii*), *B. burgdorferi sensu stricto* (*B. burgdorferi s.s.*), and *Borreliella garinii* (*B. garinii*), account for the majority of LD cases in the world (Baranton et al., 1992; Steere et al., 2016). Infection with *B. burgdorferi s.s*., the causative genospecies of LD in both the United States and Europe, often results in arthritis. In contrast, *B. afzelii* and *B. garinii*, which are other pathogenic genospecies found in Europe, predominantly cause dermatological and neurological symptoms, respectively (Van Dam et al., 1993; Steere, 2001; Steere et al., 2016).

LD is a complex, multi-systemic, and often slowly progressing illness, which is notoriously difficult to diagnose. With the exception of erythema migrans (EM), the pathognomonic but not-always-present skin lesion, other symptoms of early-stage LD are non-specific (Steere et al., 2016). During the early disseminated stage, LD patients may also develop lymphocytic meningitis, facial palsy, radiculoneuritis, and/or carditis (Pachner and Steere, 1985; Steere et al., 2016). The late disseminated stage of LD is characterized by arthritis and presents itself several months after the appearance of EM in untreated patients (Steere et al., 1987). Arthritis is less common among LD patients in Europe, where dermatological and neurological symptoms are dominant (Herzer, 1991).

LD is managed by treatment with oral antimicrobial agents over 10-28 days. In the United States, patients are treated with doxycycline, amoxicillin, or cefuroxime axetil, while azithromycin is used as the second-line antimicrobial agent (Lantos et al., 2021). For more advanced LD symptoms, like neuropathy and facial palsy, a 14-to-21-day course of intravenous ceftriaxone, cefotaxime, or penicillin is recommended. Non-steroidal anti-inflammatory drugs (NSAIDs) can be given with the antibiotics to manage the arthritis (Pachner et al., 1995; Arvikar and Steere, 2015). In some patients, arthritis, fatigue, and other symptoms can persist long after the conclusion of treatment (Shor et al., 2019).

To date, the only Food and Drug Administration-approved diagnostic tests for LD in the United States are based on serology. They are composed of highly sensitive (ELISA) and highly specific (Western blot) assays (the original technology) or two enzyme immunoassays (EIAs; the modified system) (Branda et al., 2011; Branda et al., 2017; Pegalajar-Jurado et al., 2018). However, this diagnostic system has numerous drawbacks. In addition to background seropositivity and cross-reactivity, the test sensitivity may significantly vary. In early-stage LD, for example, sensitivity for patients presenting with EM is only between 29% and 40%, though this number does increase as the infection progresses (>90%) (Hilton et al., 1999; Bacon et al., 2003; Aguero-Rosenfeld et al., 2005; Wormser et al., 2006; Molins et al., 2014; Lager et al., 2019; Horn et al., 2020). In contrast to the two-tiered system, other available diagnostic modalities (e.g., PCR, culture) are not recommended by the United States Centers for Disease Control and Prevention due to their inferior performance (Li et al., 2011; Moore et al., 2016; Lantos et al., 2021). For example, PCR was demonstrated to have a very low sensitivity (Alby and Capraro, 2015; Waddell et al., 2016; Nigrovic et al., 2019). Bacterial culture, which is considered the “gold” standard for many bacterial diseases, is time-consuming and is not often rewarding in a clinical setting (Steere, 2001; Steere et al., 2016).

Raman spectroscopy (RS) was recently explored for its diagnostic utility of LD (Farber et al., 2021). In our early proof-of-concept study, C3H mice, which is a common experimental mouse model of LD, were infected with wild-type strains of *B. burgdorferi s.s.*, 297 or B31, or an infectious but attenuated (unable to establish persistent infection) B31-derived mutant, Δ*vlsE*. The 297 and B31 strains represented two (RST1 and RST2, respectively) out of the three major classes of *B. burgdorferi s.s.* isolated across the United States (Liveris et al., 1996; Liveris et al., 1999). Blood samples drawn prior to and weekly after mouse infection for eight consecutive weeks were analyzed by a confocal RS (Farber et al., 2021). The overall results demonstrated that RS was a promising method for detecting active and past *B. burgdorferi* infection in the blood with an average true positive rate being 86-89%. The present report, which is an expansion of our previous study, has included blood samples from C3H mice infected with either *B. afzelii* or *B. garinii*, the pathogenic European genospecies, or *B. burgdorferi* N40, a strain of the third major clade of *B. burgdorferi s.s.* (RST3) in the United States. Moreover, this study involved 90 serum samples from the Lyme Disease Biobank (LDB) that originated from LD-confirmed, LD-negative, and LD-probable human patients.

## Methods

### Bacteria and culture conditions

*Borreliella* strains were grown in Barbour-Stoenner-Kelly II medium supplemented with 6% rabbit serum (referred to here as BSK-II medium; Gemini Bio-Products, CA, USA) under 2.5% CO_2_ at 35°C. To culture animal tissues, the antimicrobial cocktail composed of 0.02 mg/mL phosphomycin, 0.05 mg/mL rifampicin, and 2.5 mg/mL amphotericin B was added to BSK-II medium in order to prevent bacterial and fungal contamination.

### Mouse infection and blood sampling

A total of 20 male C3H/HeJ (C3H) mice of 4-6 weeks of age were purchased from the Jackson Laboratories (ME, USA). The mice were randomly split into four groups of five and, after a short adaption period, three groups were needle inoculated with 1.1x10^6^ cells of *B. afzelii* strain PGau (referred to here as *B. afzelii* PGau; group A), *B. garinii* subsp. bavariensis PBi (referred to here as *B. garinii* PBi; group B), or *B. burgdorferi s.s.* strain N40 (referred to here as *B. burgdorferi* N40; group C) per animal. Five other C3H mice (group D) remained uninfected and served as an age- and sex-matched control. Each mouse group was caged separately. To verify the infection in the challenged animals, their blood and other tissues were harvested and cultured in BSK-II medium as described (Rogovskyy et al., 2016). Specifically, 50 μl of blood was sampled from each mouse *via* maxillary bleed at day 7 postinfection (pi). Biopsies of ear pinnae from the *B. afzelii* PGau- and *B. garinii* PBi-infected mice were harvested at day 21 pi. Ear tissues of *B. burgdorferi* N40*-*challenged animals were sampled at 28 pi. At day 56 pi, bladder, ear pinnae, heart, and tibiotarsal joints were also harvested and cultured in BSK-II medium as detailed (Rogovskyy et al., 2016). The presence or absence of viable spirochetes was confirmed by weekly observing the cultures *via* dark-field microscopy for four weeks. At days 0, 3 (except for *B. afzelii* PGau), 7, and weekly onwards until day 56 pi, 50 μl of blood was collected from each animal *via* cheek bleed. Individual blood samples were placed in sterile Eppendorf tubes, allowed to be clotted, and then stored at −80°C until RS analysis.

### Human blood samples

Human whole blood samples (collected in EDTA tubes) were acquired from the Lyme Disease Biobank (LDB) (Horn et al., 2020). LDB is a repository of well-characterized samples, which was specifically established to facilitate research on LD and other medically important tick-borne diseases. For this study, a total of 90 blood specimens were obtained from LDB. Of them, 45 blood samples (referred to here as C samples) were acquired from LD patients, individuals enrolled with signs and symptoms of early LD, whose diagnosis was confirmed by the two-tiered testing algorithm, two positive ELISAs with EM > 5 cm, IgG seroconversion at the second draw, LD-positive culture and/or PCR (**Table S1**). The 30 serology-negative endemic control (EC) samples were obtained from healthy individuals without a history of LD or other tick-borne infections, who tested negative by all serologic tests. The 15 LD probable (P) samples were acquired from LD patients, who had been clinically diagnosed with LD (all developed EM lesions of > 5 cm) but were negative by the two-tiered testing algorithm (**Table S1**) (Horn et al., 2020).

### Raman spectroscopy

To prepare them for RS, blood samples were melted by hand and then vortexed before 50 µL were spread in a thin layer on a foil-wrapped microscope slide. The aluminum foil was flattened with a razor blade before wrapping. Samples were dried for 30 minutes in a fume hood before scanning. Spectra were obtained using a home-built confocal Raman microscope with a 785 nm continuous-wave laser (Necsel, NJ, USA) with a laser power of ~8.0 mW. The laser beam was guided into the inverted microscope (Nikon TE-2000 U) *via* a set of mirrors and passed through a 50/50 beam splitter. The laser was focused on the dried blood surface through a 20X Nikon objective lens (NA = 0.45). The scattered light was directed through an LP02-785RE-25 long-pass filter (Semrock, NY, USA) to filter Rayleigh scattering, then passed to an IsoPlane SCT 320 spectrograph (Princeton Instruments, NJ, USA) equipped with a 600 groove/mm grating blazed at 750 nm. The light was then sent to a PIXIS:400BR CCD (Princeton Instruments). The sample was moved relative to the incident laser beam using an H117P2TE (Prior, MA, USA) motorized stage, controlled by a Prior Proscan II. A total of 19,235 Raman spectra were obtained throughout the experiment (spectral acquisition time was 30 s). During preprocessing, the spectra’s baseline was flattened and the area under the curve was normalized. The following spectra were what the partial least squares discriminant analysis (PLS-DA) model analyzed.

### Statistical analysis

Analysis of variance (ANOVA) was performed in MATLAB or the MATLAB add-on PLS_Toolbox (Eigenvector Research, Inc., WA, USA). Raw spectra were first averaged in groups of five to reduce noise. These averaged spectra were then baseline-corrected using the Automatic Weighted Least Squares algorithm with a 6^th^ order polynomial then normalized to the total area under the curve of the spectrum.

Partial least squares-discriminant analysis (PLS-DA) was used to differentiate between timepoints after infection for each genospecies and the uninfected control. Then, 45 binary models per genospecies/uninfected control were generated with the exception of *B. afzelii* PGau because day 3 postinfection samples were not available. Binary models were also constructed comparing each time point for each of the genospecies to all the control spectra. The best performing model was then recorded, based on the highest Matthew’s Correlation Coefficient (MCC), which has been found to be a more robust way of evaluating binary models than either F1 score or accuracy because it takes into account all four values in the confusion matrix (Chicco and Jurman, 2020; Yao and Shepperd, 2020; Chicco et al., 2021). In order to determine whether the peaks were significantly different, Kruskal-Wallis one-way ANOVA was conducted on the peaks used by the PLS-DA model as shown by the loading plots from the PLS_toolbox. Differences were considered statistically significant at a P value of 0.05. *Post-hoc* Tukey HSD tests were performed to generate 95% confidence intervals.

## Results

### Mouse infection

Although all 15 blood samples taken at day 7 pi were culture-negative, the successful infection of three groups of C3H mice (A, B, and C) was confirmed by culture-positive results of ear pina biopsies harvested at day 21 (*B. afzelii* PGau and *B. garinii* PBi) or 28 (*B. burgdorferi* N40) pi. The presence of long-term infection was verified by positive cultures of mouse tissues harvested at day 56 pi for all *B. afzelii* PGau- and *B. burgdorferi* N40-infected mice (**Table 1**). However, a long-term infection by *B. garinii* PBi was only detected in two out of 5 mice as shown by positive cultures of their ear tissues. The lack of culture-detectable *B. garinii* PBi may suggest that the three mice had either cleared the infection, or the numbers of spirochetes were low (under the threshold of detection by culture) at the time the mouse tissues were collected.

**Table 1:** Culture results of tissues harvested from *Borreliella*-infected C3H mice.

Mouse groups	Challenge with	No. of culture-positive mice/total no. of mice tested					
		Blood (day 7 pi)	Ear pinna biopsy (day 21* pi)	Bladder (day 56 pi)	Ear pinna biopsy (day 56 pi)	Joint (day 56 pi)	Heart (day 56 pi)
**A**	*B. afzelii* PGau	0/5	5/5	5/5	5/5	5/5	5/5
**B**	*B. garinii* PBi	0/5	5/5	0/5	2/5	0/5	0/5
**C**	*B. burgdorferi* N40	0/5	5/5	4/5	5/5	4/5	4/5

*The ear pinna biopsies of mice infected with *B. burgdorferi* N40 were sampled at 28 postinfection (pi).

### The analysis of mouse blood by Raman spectroscopy

**Figure 1:**
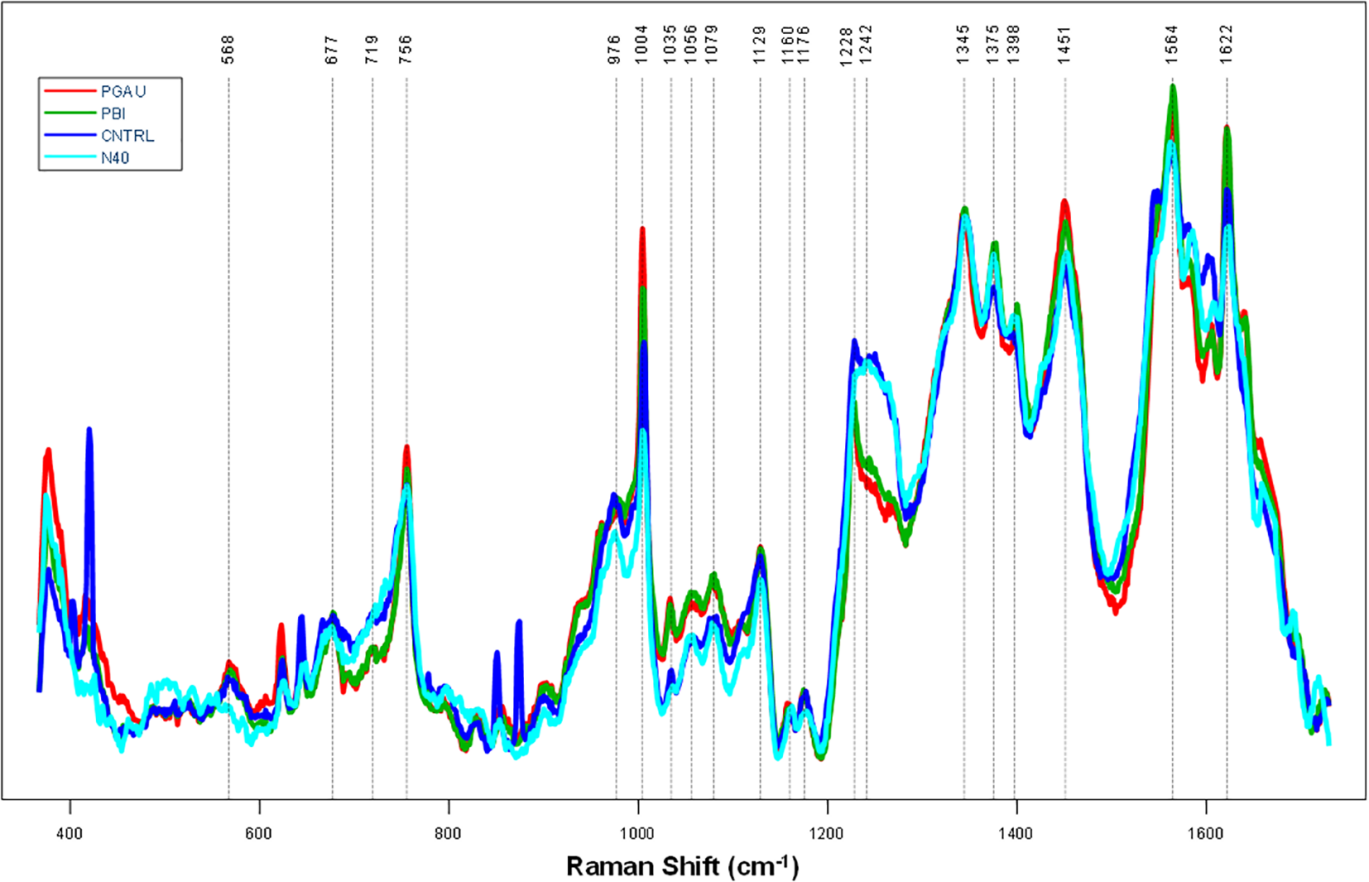
The average preprocessed (Automatic Whitaker Filter baselined and area normalized) Raman spectra for blood samples taken from *Borreliella*-infected C3H mice at day 56 postinfection. C3H mice (5 animals per group) were infected with *B afzelii* PGau (PGAU), *B garinii* PBi (PBI), or *B burgdorferi* N40 (N40). One group of mice remained uninfected and served as a control (CNTR).

Overall, the Raman spectra collected from blood of mice infected with *B. afzelii* PGau, *B. garinii* PBi, or *B. burgdorferi* N40 exhibited vibrational bands that could be assigned to proteins, heme, sugars, carotenoids, and other biomolecules (**Figure 1** and **Table 2**). Regardless of the *Borreliella* genospecies and time points the blood was collected at, only small changes in the relative intensities of the vibrational bands were observed.

**Table 2:** Assignments of vibrational bands for the Raman spectra acquired from blood samples of *Borreliella*-infected C3H mice.

Band (cm^-1^)	Assignment
562	Fe-O_2_ stretch (heme) (Casella et al., 2011)
676	Pyrrole symmetric bending (Heme) (Casella et al., 2011)
719	C-C-O related to glycosidic ring skeletal deformations (Krimmer et al., 2019)
752	Protein (Pichardo-Molina et al., 2007), Heme ring breathing (Casella et al., 2011)
962	Associated with alpha CH of porphyrin ring (Lemler et al., 2014)
1002	Phenylalanine ring breathing (Khan et al., 2016), CH_3_ in-plane rocking of polyenes (Khan et al., 2016)
1126	C – C stretching (Khan et al., 2016)
1172	Trp, Phe (Pichardo-Molina et al., 2007)
1226	CH Bending (Heme) (Casella et al., 2011)
1249	*meso* CH of porphyrin ring (Lemler et al., 2014)
1275	Lipids, Amide III (Pichardo-Molina et al., 2007)
1308	*meso* CH of porphyrin ring (Lemler et al., 2014)
1340	Trp, Adenine, Lipids (Pichardo-Molina et al., 2007)
1376	Pyrrole ring (Atkins et al., 2017)
1447	CH_2_ (Khan et al., 2016)
1462	CH_2_, CH_3_ (Başar et al., 2012)
1516	C=C (Khan et al., 2016)
1562	Conjugated CC stretching (heme) (Casella et al., 2011)
1579	C – C stretching (Khan et al., 2016)
1604	Aromatic ring (Khan et al., 2016)
1622	Aromatic ring (Khan et al., 2016)
1657	Amide I, C=C (Khan et al., 2016; Farber et al., 2020)
1681	Amide I (Başar et al., 2012), carboxylic acids

**Figure 2:**
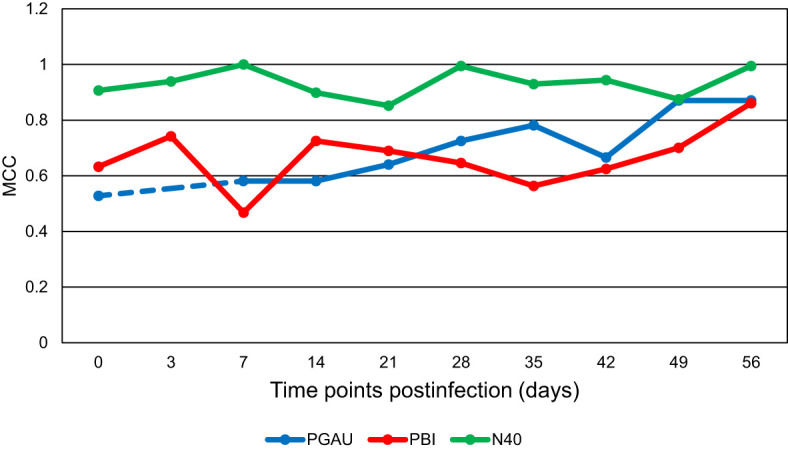
The Matthew’s Correlation Coefficient (MCC) for the binary model comparing the uninfected control to each of the three *Borreliella* genospecies over time. Mouse blood was sampled from C3H mice prior to their infection and postinfection with *B afzelii* PGau (PGAU), *B garinii* PBi (PBI), or *B burgdorferi* N40 (N40). The blood was also collected from age- and sex-matched uninfected (control) C3H mice. The dashed line indicates that the data are missing.

By using our previously developed PLS-DA method (Farber et al., 2021), the prediction accuracy for the collected spectra was determined. As demonstrated by the MCC (**Figure 2**), the ability of the model to detect *B. garinii* PBi infection in C3H mice at day 7 pi was only 46.8%. However, the MCC increased with progression of the mouse infection, reaching 86.1% at day 56 pi (**Figure 2**). Similar results were obtained for the accuracy of detection of mouse infection with *B. afzelii* PGau. Specifically, the diagnostic ability of the model increased from 58% for day 7 to 78% for day 35 pi. Surprisingly, there was a drastic decrease in the detection accuracy for day 42 pi (67%), whereas the MCC for days 49 and 56 pi was 87% (**Figure 2**). In contrast, the model, which was used to differentiate between the control blood from uninfected mice and the blood sampled from *B. burgdorferi* N40-infected animals, showed no clear trend with MCC values being consistently high for all time points, including day 0 (pre-infection).

**Figure 3:**
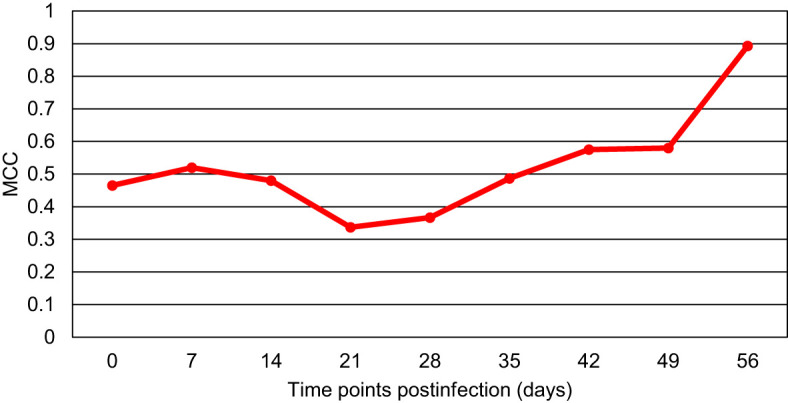
The Matthew’s Correlation Coefficient (MCC) for the binary model comparing blood samples of all *Borreliella*-infected mice to blood samples of all uninfected controls for each time point. Mouse blood was sampled from C3H mice prior to their infection and postinfection with *B afzelii* Pgau (PGAU), *B garinii* PBi (PBI), or *B burgdorferi* N40 (N40). The blood was also collected from age- and sex-matched uninfected (control) C3H mice.

**Figure 4:**
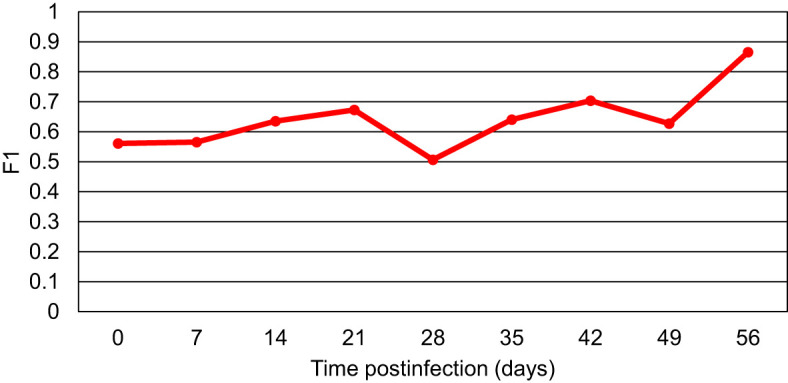
The F1 scores for the models comparing blood samples of all *Borreliella*-infected mice to blood samples of all uninfected controls for each time point. Mouse blood was sampled from C3H mice prior to their infection and postinfection with *B afzelii* Pgau (PGAU), *B garinii* PBi (PBI), or *B burgdorferi* N40 (N40). The blood was also collected from age- and sex-matched uninfected (control) C3H mice.

The acquired RS data were also combined to generate the binary model that would differentiate between the blood from all *Borreliella*-infected mice and the blood of uninfected control animals for each time point (**Figure 3**). The result showed that the only model with high predictability was the model for day 56 pi (MCC of 89%). The MCC values for the models of the other time points were consistently low (MCC of 34-58%). For the day 56 model, the odds ratio was calculated to be 3069.3 (z=5.584, p<0.0001) with the accuracy, sensitivity, and specificity being 96%, 94% and 100%, respectively. Similarly, as calculated by F1, the models of the earlier time points demonstrated a low diagnostic capacity (51-70%), whereas the day 56 model more accurately differentiated all *Borreliella*-infected mice from the respective uninfected controls (87%; **Figure 4**).

In order to examine the capacity of RS to differentiate different timepoints of mouse infection, the Raman spectra acquired from weekly blood samples were compared within each genospecies. The overall results demonstrated that MCC ranges were 66-100%, 68-100%, and 80-100% for *B. afzelii* PGau, *B. garinii* PBi, and *B. burgdorferi* N40, respectively (**Tables 3**-**5**). The frequency at which the timepoints were differentiated with the highest accuracy (MCC of 100%) was much higher for blood samples obtained from *B. burgdorferi* N40- or *B. garinii* PBi-infected mice (53% and 31%, respectively). In contrast, when weekly samples from *B. afzelii* PGau-infected animals were compared, only one comparison (day 0 vs 42 pi) had an MCC of 100%. However, the MCC values of 90-99% were more frequently observed for infection with *B. afzelii* PGau (58%; **Table 3**) than that of *B. garinii* PBi (42%; **Table 4**) or *B. burgdorferi* N40 (33%; **Table 5**). Overall, the timepoints of mouse infection were differentiated with the highest accuracy (MCC of 90-100%) for *B. burgdorferi* N40 (87%) followed by *B. garinii* PBi (73%) and *B. afzelii* PGau (61%).

**Table 3:** The accuracy of identification of *B. afzelii* PGau infection in C3H mice at different time points postinfection as measured by the Matthew’s Correlation Coefficient.

Days post infection	7	14	21	28	35	42	49	56
**0**	0.960*	0.980	0.800	0.931	0.990	1.000	0.990	0.930
**7**	n/a**	0.910	0.950	0.980	0.990	0.980	0.990	0.980
**14**	n/a	n/a	0.860	0.960	0.910	0.890	0.970	0.830
**21**	n/a	n/a	n/a	0.702	0.800	0.700	0.960	0.712
**28**	n/a	n/a	n/a	n/a	0.860	0.921	0.680	0.863
**35**	n/a	n/a	n/a	n/a	n/a	0.931	0.660	0.790
**42**	n/a	n/a	n/a	n/a	n/a	n/a	0.942	0.800
**49**	n/a	n/a	n/a	n/a	n/a	n/a	n/a	0.901

*Matthew’s Correlation Coefficient value.

**n/a denotes non-applicable.

**Table 4:** The accuracy of identification of *B. garinii *PBi infection in C3H mice at different time points postinfection as measured by the Matthew’s Correlation Coefficient.

Days post infection	3	7	14	21	28	35	42	49	56
**0**	0.771*	0.762	0.990	1.000	1.000	1.000	0.990	1.000	1.000
**3**	n/a**	0.771	0.569	0.750	0.871	0.980	0.852	1.000	0.990
**7**	n/a	n/a	0.854	0.891	0.940	0.980	0.940	1.000	1.000
**14**	n/a	n/a	n/a	0.682	0.921	0.980	0.990	1.000	0.990
**21**	n/a	n/a	n/a	n/a	0.940	0.990	0.950	0.970	1.000
**28**	n/a	n/a	n/a	n/a	n/a	0.920	0.890	1.000	1.000
**35**	n/a	n/a	n/a	n/a	n/a	n/a	0.920	0.980	1.000
**42**	n/a	n/a	n/a	n/a	n/a	n/a	n/a	0.980	1.000
**49**	n/a	n/a	n/a	n/a	n/a	n/a	n/a	n/a	0.713

*Matthew’s Correlation Coefficient value.

**n/a denotes non-applicable.

**Table 5:** The accuracy of identification of *B. burgdorferi* N40 infection in C3H mice at different time points postinfection as measured by the Matthew’s Correlation Coefficient.

Days post infection	3	7	14	21	28	35	42	49	56
**0**	0.881*	1.000	1.000	1.000	1.000	0.990	1.000	0.940	1.000
**3**	n/a**	0.980	0.862	0.980	0.885	0.961	0.797	0.960	0.980
**7**	n/a	n/a	1.000	0.922	1.000	1.000	1.000	1.000	1.000
**14**	n/a	n/a	n/a	0.851	0.950	0.970	1.000	1.000	1.000
**21**	n/a	n/a	n/a	n/a	0.950	0.950	1.000	0.990	1.000
**28**	n/a	n/a	n/a	n/a	n/a	0.980	0.875	1.000	0.990
**35**	n/a	n/a	n/a	n/a	n/a	n/a	1.000	1.000	1.000
**42**	n/a	n/a	n/a	n/a	n/a	n/a	n/a	1.000	1.000
**49**	n/a	n/a	n/a	n/a	n/a	n/a	n/a	n/a	1.000

**Matthew’s Correlation Coefficient value.

*n/a denotes non-applicable.

### The analysis of human blood by Raman spectroscopy

**Figure 5:**
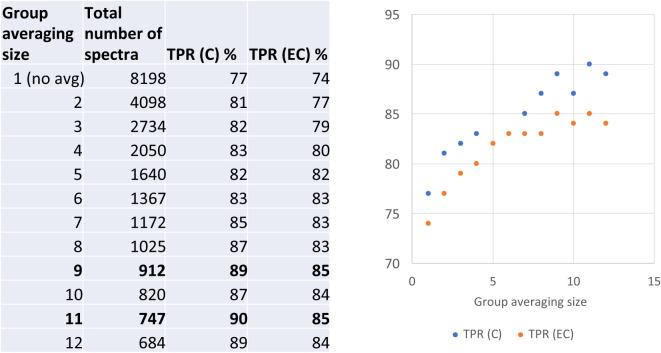
The accuracy of differentiation between serologically negative (EC) and LD-confirmed (C) groups of human blood samples when averaged in different sized groups. TPR denotes a true positive rate.

To further explore the diagnostic capacity of RS, human blood samples acquired from LDB were analyzed (Horn et al., 2020). A total of 45 C, 30 EC, and 15 P samples were included in this study. As a result of the RS analysis, 8198 spectra were collected from the 90 human blood samples. In order to determine the capacity of RS to differentiate between the C and EC samples, the respective spectra were analyzed and compared by our previously developed PLS-DA model (Farber et al., 2021). The results demonstrated that the PLS-DA model identified the healthy individuals (the EC samples) and LD patients (the C samples) with the accuracy of 74% and 77%, respectively (**Figure 5**). To increase the PLS-DA accuracy, signal-to-noise quality of spectra was improved by averaging the spectra. When 9 or 11 spectra were averaged together in one group, the accuracy of identifying blood samples of the healthy individuals and LD patients increased to 85% of and 90%, respectively (**Figure 5**). The improved PLS-DA model was further applied to analyze the 15 P samples (the patients who were clinically diagnosed with EM > 5 cm but tested negative by the two-tiered testing algorithm). Based on their matching scores, samples P11, P12, and P13 were assigned to the C sample group with a high probability (91%, 98%, and 87%, respectively) (**Table 6**). In contrast, samples P1-P5 had low MCC values (0-1%) and, therefore, were assigned to the EC sample group (**Table 6**). Samples P6-P10 could be assigned to the C sample group with MCC values of 66-79%. The MCC values of P14 and P15 were 33% and 44%, respectively, making their group assignment undetermined (**Table 6**).

**Table 6:** The Raman spectroscopy test results of LD probable human blood samples.

	Human blood samples														
	P1	P2	P3	P4	P5	P6	P7	P8	P9	P10	P11	P12	P13	P14	P15
**MCC**	0.007*	0	0	0.02	0.04	0.66	0.74	0.58	0.79	0.71	0.91	0.98	0.87	0.33	0.44
**Result interpretation**	N**	N	N	N	N	P**	P	P	P	P	P	P	P	N	N

*MCC denotes the Matthew’s Correlation Coefficient.

**N and P denote negative and positive results, respectively.

After all models were created, ANOVA was performed on the pre-processed spectra in order to detect any significant differences in the intensity of Raman scattering for various peaks. Specifically, the analysis involved a total of 15 peaks, which were most used by each model. To differentiate between the three *Borreliella* genospecies, loading plots outputted by the PLS-DA were utilized, and the following peaks were identified: 420, 643, 745, 874, 973, 1008, 1249, 1351, 1394, 1426, 1454, 1540, 1592, 1627, and 1782 cm^-1^. **Tables S2** through **S5** show the results of the ANOVA and *post-hoc* Tukey HSD test (significant vs non-significant) for each peak and model tested. The ANOVA results demonstrated that each generated model had at least 9 significantly different peaks, and that, for four models, significant difference was observed for all 15 peaks (**Tables S2-S5**). Together, the results indicated that the models generated in this study were statistically significant.

## Discussion

The results of the present study were consistent with our previous findings, confirming that RS coupled with chemometrics could identify *Borreliella*-induced infection in C3H mice with high accuracy (Farber et al., 2021). Although RS was previously shown to differentiate the blood from *Borreliella*-infected mice from pre-infection samples after the infection had been cleared (Farber et al., 2021), it is still possible that low numbers of wild-type spirochetes could have been present in the mouse blood samples tested in the current study. Overall, the present data suggested that RS had sufficient discriminating capacity to differentiate between early and late stages (timepoints) of LD infection in the mouse model. The combined results of our studies also showed that mouse infection by a representative strain (297, B31, or N40) of the three major clades of *B. burgdorferi s.s.* (RST1, RST2, and RST3, respectively) in the United States (Liveris et al., 1996; Liveris et al., 1999) could be equally detected by RS. In addition to *B. burgdorferi s.s.*, the current data demonstrated that RS could also identify mouse infection of additional genospecies of *B. burgdorferi s*.*l.*, *B. afzelii* and *B. garinii*, though the accuracy of their detection was only high for the late stage (day 56 pi). The reason for the latter is not obvious and requires further investigation. Overall, blood sampled from *Borreliella*-infected mice was detected with 96% accuracy, 94% sensitivity, and 100% specificity. Similarly, human blood samples were analyzed with 88% accuracy, 85% sensitivity, and 90% specificity. The observed differences were most likely due to variability that human samples inevitably introduced (e.g., bacterial strain, infectious dose, stage of infection, patient immune status, treatment) as opposed to samples that were generated from inbred mice under the well-controlled study. This human sample variability may also account for different MCC values obtained from the P samples. Collectively, the present results indicate that RS could be further explored as a follow-up assay for those patients, who are clinically diagnosed as LD-positive (due to their erythema migrans of > 5 cm) and yet test negative by the existing two-tier serologic testing.

In addition to our published study (Farber et al., 2021), two other subsequent investigations that had also examined the RS capacity for LD diagnostics were recently published (Senger et al., 2022; Tabb et al., 2022). One study compared Raman spectra acquired from urine samples of 30 serology-confirmed LD patients against urine spectra from 235 LD-negative (healthy) individuals, 362 patients with end-stage kidney disease, and 17 bladder cancer patients (Senger et al., 2022). As a result, the study demonstrated that RS could distinguish the LD-confirmed patients from the other groups with 87-88% accuracy, 83-87% sensitivity, and 87-90% specificity (Senger et al., 2022). These assay characteristics were almost identical to those of the present RS analysis of the human blood samples.

The other recent investigation has examined the capacity of surface enhanced Raman scattering (referred to here as SERS) to directly detect *Borreliella* spirochetes in serum samples of LD pediatric patients (Tabb et al., 2022). Outer surface protein A (OspA), a surface lipoprotein whose function is to mediate the attachment of spirochetes to the tick midgut (Fingerle et al., 1995; Schwan et al., 1995), was chosen as a target of DNA aptamers. By testing 24 LD-negative and 23 LD-positive samples, this approach showed higher accuracy (94%), sensitivity (91%) and selectivity (96%) than the presently and previously utilized indirect RS-based detection methods (Senger et al., 2022). However, the results of this OspA aptamer-based SERS are quite surprising. The seminal study clearly demonstrated that OspA is not expressed during mammalian (mouse) infection (Ohnishi et al., 2001). Thus, in sera of LD patients, the chosen OspA target is either absent, or at best is present at very low levels. The latter was only shown by a single study that involved sera from a very limited number of patients (n=3) (Cheung et al., 2015). Furthermore, in addition to its complicated and time consuming sample preparation (Tabb et al., 2022), it is unknown how serum handling, freeze-thaw, and long-term storage affect the stability of peptide targets for SERS (Dufresne et al., 2017).

Overall, there are two overarching caveats of the present and above-mentioned studies (Farber et al., 2021; Senger et al., 2022; Tabb et al., 2022). First, the clinical samples have not been tested in a blind manner. Second, the studies did not include samples from LD-negative patients, who would be diagnosed with other (tick-borne) infectious diseases. An adequate evaluation of RS specificity is, therefore, highly warranted. To achieve this, future studies should include blood samples both from mice experimentally infected with various pathogens and humans with confirmed diagnosis of the respective diseases.

It should be noted that the current study has other limitations. First, due to a large scale of work, the RS data were collected by different persons, which unavoidably introduced some variability. To minimize the latter, the process of RS scanning could be automated (Lopez-Reyes and Rull Pérez, 2017; O’dwyer et al., 2021). Second, as with many biological materials, the Raman spectra collected from the blood were relatively noisy, most likely due to such factors as fluorescence and chemical complexity of biological samples (Lin et al., 2012; Leon Bejarano et al., 2017; Barton and Hennelly, 2018; Jahn et al., 2021). Although the inter-scanner variability and noise were reduced through the averaging of spectra, the issue still persisted in the present study. To further reduce the noise, vials composed of quartz could be used for the direct scanning of blood. A quartz vial would allow for a thicker layer of blood compared to the foil-wrapped slide used in the present study and would avoid the issue of interference. It was shown that quartz does not exhibit Raman fluorescence or heavy Raman scattering beyond 500 cm^-1^ Raman shift (Tuschel, 2016). Third, some peaks may have hindered an accurate identification of component compounds. For example, the peak at 1657 cm^-1^ could represent vibrations from an amide bond, characteristic of all proteins, or an alkene group in unsaturated fatty acids (Khan et al., 2016; Farber et al., 2020; Farber et al., 2021). To accurately identify compounds and their functional groups, high performance liquid chromatography (HPLC) could potentially be applied (Dou et al., 2021; Higgins et al., 2022). Previous utilization of HPLC coupled to mass spectrometry has successfully identified changes in the metabolic profile of the blood from patients diagnosed with early-stage LD (Molins et al., 2015). A vast majority of the molecular species identified in LD patients’ blood were lipids or their derivatives (e.g., cholesterol, cholesteryl acetate, diacylglycerol, phospholipids, sphingolipids, triglycerides) (Molins et al., 2015).

Together, the results of the current and other studies (Farber et al., 2021; Senger et al., 2022; Tabb et al., 2022) are highly encouraging, as they consistently suggested that RS could potentially be utilized as a diagnostic test for LD patients. The transitioning from using a large RS instrument (confocal Raman spectrometer) used in this study to a hand-held, easy-to-operate Raman spectrometer would be another next step towards utilization of RS for LD diagnostics. Given the portability and easy sample preparation process (the spreading of blood onto a foil-wrapped microscope slide), the testing of blood samples by RS may ultimately be deployed in medically underserved areas that often lack access to diagnostic laboratories.

## Data availability statement

The raw data supporting the conclusions of this article will be made available by the authors, without undue reservation.

## Ethics statement

The mouse experimental procedures were approved by the Institutional Animal Care and Use Committee of Texas A&M University. All experiments were performed in accordance with Public Health Service (PHS) Policy on Humane Care and Use of Laboratory Animals (2002), Guide for the Care and Use of Agricultural Animals in Research and Teaching (2010), and Guide for the Care and Use of Laboratory Animals (2011). Institutional review board (IRB) approval was obtained for each site through the LDB sponsor protocol (Advarra IRB protocol Pro00012408) or the site’s local IRB.

## Author contributions

NG was involved in data collection, analysis, writing, and project management. TD was involved in data analysis. RM was involved in the collection of spectra from human blood. SH and KM were involved in data collection. EH provided resources and was involved in the study design and edited the manuscript. AR concepted and developed the study and wrote the manuscript. DK concepted and developed the study, oversaw the project, and edited the manuscript. All authors contributed to the article and approved the submitted version.

## Funding

Funding for this study was provided by the 2020 Bay Area Lyme Foundation’s Emerging Leader Fund, 2022 Bay Area Lyme Foundation award, and the Texas A&M AgriLife Research Insect Vector Disease Seed Grant.

## Acknowledgments

The authors would like to thank Maliha Batool for her help with the mouse experiments, and Bridget Bedore, Natalia Ayala, Stephen Parlamas and Dillon Humpal for their assistance with the acquisition of Raman spectra. We are grateful to Troy Bankhead, Jenifer Coburn, Richard Marconi, Ulrike Munderloh, and Jon Skare for providing the bacterial strains for this study.

## Conflict of interest

The authors declare that the research was conducted in the absence of any commercial or financial relationships that could be construed as a potential conflict of interest.

## Publisher’s note

All claims expressed in this article are solely those of the authors and do not necessarily represent those of their affiliated organizations, or those of the publisher, the editors and the reviewers. Any product that may be evaluated in this article, or claim that may be made by its manufacturer, is not guaranteed or endorsed by the publisher.
