# Supplementary material for: Testing Raman spectroscopy as a diagnostic approach for Lyme disease patients
Source: Front Cell Infect Microbiol. 2022 Oct 27;12:1006134. doi: 10.3389/fcimb.2022.1006134 (PMC9647194; doi:10.3389/fcimb.2022.1006134)
Supplement: Supplementary file 2 [file Table_2.docx]

**SUPPLEMENTAL MATERIALS**

Table S2. The ANOVA results of the peaks used by the models comparing blood from *Borreliella*-infected C3H mice to blood from (uninfected) controls for each time point postinfection.

| **Raman Shift (cm^-1^)** | **Time point postinfection (days)** | | | | | | | | |
| --- | --- | --- | --- | --- | --- | --- | --- | --- | --- |
|  | **0** | **7** | **14** | **21** | **28** | **35** | **42** | **49** | **56** |
| **420** | N* | Y | Y | Y | Y | Y | Y | Y | Y |
| **643** | Y** | Y | Y | Y | Y | Y | Y | Y | Y |
| **745** | Y | Y | Y | Y | Y | Y | Y | Y | Y |
| **874** | Y | Y | Y | Y | Y | Y | Y | Y | Y |
| **973** | N | Y | Y | Y | Y | Y | Y | Y | Y |
| **1008** | Y | Y | Y | N | N | N | Y | Y | Y |
| **1249** | Y | Y | Y | Y | Y | Y | Y | Y | Y |
| **1351** | N | Y | Y | Y | Y | Y | N | Y | N |
| **1394** | Y | Y | Y | Y | N | Y | Y | Y | N |
| **1426** | Y | Y | N | Y | Y | Y | Y | Y | Y |
| **1454** | Y | Y | Y | N | Y | Y | Y | Y | Y |
| **1540** | Y | N | Y | N | Y | Y | Y | Y | Y |
| **1592** | Y | Y | Y | Y | Y | Y | Y | N | Y |
| **1627** | Y | Y | Y | Y | Y | Y | Y | Y | N |
| **1782** | Y | Y | Y | Y | Y | N | N | Y | Y |
| **Total number of significant peaks** | 12 | 14 | 14 | 12 | 13 | 13 | 13 | 14 | 12 |

*N indicates the peaks that are not statistically significant.

**Y indicates statistically significant peaks.

**Table S3.** **The ANOVA results of the peaks used by the models comparing blood from B. afzelii PGau-infected C3H mice to blood from (uninfected) controls for each time point postinfection.**

| **Raman Shift (cm^-1^)** | **Time point postinfection (days)** | | | | | | | | |
| --- | --- | --- | --- | --- | --- | --- | --- | --- | --- |
|  | **0** | **7** | **14** | **21** | **28** | **35** | **42** | **49** | **56** |
| **420** | Y* | Y | Y | Y | Y | Y | Y | Y | Y |
| **643** | Y | Y | Y | Y | Y | Y | Y | Y | Y |
| **745** | Y | Y | Y | Y | Y | Y | Y | N | Y |
| **874** | Y | Y | Y | Y | Y | Y | Y | Y | Y |
| **973** | N** | N | Y | Y | Y | Y | Y | Y | Y |
| **1008** | Y | Y | Y | Y | Y | N | Y | Y | Y |
| **1249** | Y | Y | Y | Y | Y | Y | Y | Y | Y |
| **1351** | Y | Y | N | Y | Y | Y | Y | Y | Y |
| **1394** | N | Y | Y | Y | N | Y | Y | Y | N |
| **1426** | N | Y | Y | Y | N | Y | Y | N | N |
| **1454** | Y | Y | Y | Y | Y | Y | Y | Y | Y |
| **1540** | Y | Y | Y | Y | Y | Y | Y | N | Y |
| **1592** | Y | Y | Y | Y | Y | Y | Y | Y | Y |
| **1627** | Y | Y | Y | Y | Y | N | Y | Y | Y |
| **1782** | Y | Y | Y | Y | Y | Y | Y | Y | Y |
| **Total number of significant peaks** | 12 | 14 | 14 | 15 | 13 | 13 | 15 | 12 | 13 |

*Y indicates statistically significant peaks.

**N indicates the peaks that are not statistically significant.

**Table S4. The ANOVA results of the peaks used by the models comparing blood from B. garinii PBi-infected C3H mice to blood from (uninfected) controls for each time point postinfection.**

| **Raman Shift (cm-1)** | **Time point postinfection (days)** | | | | | | | | | |
| --- | --- | --- | --- | --- | --- | --- | --- | --- | --- | --- |
|  | **0** | **3** | **7** | **14** | **21** | **28** | **35** | **42** | **49** | **56** |
| **420** | Y* | N | Y | N | Y | N | Y | Y | Y | Y |
| **643** | N** | Y | Y | Y | Y | Y | Y | Y | Y | Y |
| **745** | Y | Y | Y | Y | Y | N | Y | Y | Y | Y |
| **874** | Y | Y | Y | Y | Y | Y | Y | Y | Y | Y |
| **973** | N | Y | N | N | Y | Y | N | N | Y | Y |
| **1008** | Y | Y | N | Y | Y | N | N | Y | N | N |
| **1249** | Y | Y | N | Y | Y | Y | Y | Y | Y | Y |
| **1351** | N | N | Y | N | Y | N | Y | Y | Y | N |
| **1394** | N | Y | Y | N | Y | N | Y | Y | Y | N |
| **1426** | N | Y | Y | N | Y | Y | Y | Y | Y | N |
| **1454** | Y | Y | N | Y | N | Y | Y | Y | Y | Y |
| **1540** | N | N | N | Y | Y | Y | N | Y | Y | Y |
| **1592** | Y | Y | Y | Y | Y | Y | Y | Y | Y | Y |
| **1627** | Y | N | Y | Y | N | Y | Y | N | Y | N |
| **1782** | Y | N | Y | Y | Y | N | Y | Y | Y | Y |
| **Total number of significant peaks** | 9 | 10 | 10 | 10 | 13 | 9 | 12 | 13 | 14 | 10 |

*Y indicates statistically significant peaks.

**N indicates the peaks that are not statistically significant.

**Table S5.** **The ANOVA results of the peaks used by the models comparing blood from B. burgdorferi N40-infected C3H mice to blood from (uninfected) controls for each time point postinfection.**

| **Raman Shift (cm^-1^)** | **Time point postinfection (days)** | | | | | | | | | |
| --- | --- | --- | --- | --- | --- | --- | --- | --- | --- | --- |
|  | **0** | **3** | **7** | **14** | **21** | **28** | **35** | **42** | **49** | **56** |
| **420** | Y* | N | Y | Y | Y | Y | Y | Y | Y | Y |
| **643** | Y | Y | Y | Y | Y | Y | Y | Y | Y | Y |
| **745** | Y | Y | Y | Y | N | Y | Y | Y | Y | N |
| **874** | Y | Y | Y | Y | Y | Y | Y | Y | Y | Y |
| **973** | N** | Y | Y | Y | Y | Y | Y | Y | Y | Y |
| **1008** | Y | Y | N | N | Y | Y | Y | Y | Y | Y |
| **1249** | Y | Y | N | N | N | N | Y | Y | Y | N |
| **1351** | Y | Y | Y | Y | N | Y | N | Y | Y | N |
| **1394** | Y | N | Y | Y | Y | Y | Y | Y | Y | Y |
| **1426** | Y | Y | Y | Y | Y | Y | Y | Y | Y | Y |
| **1454** | Y | Y | N | Y | Y | Y | Y | Y | N | Y |
| **1540** | Y | Y | Y | Y | Y | Y | Y | Y | Y | Y |
| **1592** | Y | N | Y | Y | Y | Y | Y | Y | Y | Y |
| **1627** | Y | N | Y | N | Y | Y | Y | Y | Y | Y |
| **1782** | Y | Y | Y | Y | Y | Y | Y | Y | Y | Y |
| **Total number of significant peaks** | 14 | 11 | 12 | 12 | 12 | 14 | 14 | 15 | 14 | 12 |

*Y indicates statistically significant peaks.

**N indicates the peaks that are not statistically significant.
